# Correlations of serum cystatin C and glomerular filtration rate with vascular lesions and severity in acute coronary syndrome

**DOI:** 10.1186/s12872-017-0483-8

**Published:** 2017-01-31

**Authors:** Jinjin Zhang, Xianhao Wu, Peizhen Gao, Pingping Yan

**Affiliations:** 1 Department of Cardiology, North Huashan Hospital of Fudan University, Shanghai, 201907 People’s Republic of China; 2 Department of Cardiology, Tongren Hospital, Shanghai, 200050 People’s Republic of China; 3 Department of Beixinjing Community Health Center, Shanghai, 200335 People’s Republic of China

**Keywords:** Acute coronary syndrome, Serum cystatin C, Glomerular filtration rate, Correlation study

## Abstract

**Background:**

The aim of this study was to evaluate the predictive value of cystatin C (CysC) and estimated glomerular filtration rate (eGFR) regarding vascular lesions and their severity in patients with acute coronary syndrome (ACS).

**Methods:**

According to the results of coronary angiography, 195 ACS patients were divided into a single-vascular-lesion group (91 cases), a dual-vascular-lesion group (67 cases), and a multiple-vascular-lesion group (37 cases) to assess the severity of coronary artery disease according to Gensini scores and to analyze the correlations of CysC and eGFR level with vascular lesions and severity in ACS patients.

**Results:**

Intergroup comparisons of univariate and multivariate regression analyses showed that CysC was positively correlated with vascular lesions (*P* < 0.05), but eGFR showed no correlation. Regarding the severity of vascular lesions, CysC was positively correlated with Gensini score (Pearson’s correlation coefficient *r* = 0.1811, *P* < 0.05), but eGFR was not correlated (*P* > 0.05).

**Conclusions:**

Serum CysC levels could reflect the severity of vascular lesions in ACS patients, and a high CysC level had predictive value regarding the severity of vascular lesions in ACS.

## Background

With changes in lifestyles and diet structures in China, current heart disease spectra have changed, and coronary heart disease (CHD) has become the most common cardiac disease. Meanwhile, with the acceleration of the aging process, the morbidity and mortality of CHD are exhibiting a rapidly increasing trend, and it has become a major chronic noncommunicable disease severely harming public health. Acute coronary syndrome (ACS), characterized by high incidence, high mortality, and high recurrence, is currently one of the diseases that is a serious hazard to human health. ACS includes ST segment-elevated myocardial infarction (STEMI), non-ST segment-elevated myocardial infarction (NSTEMI), and unstable angina. Different forms of ACS share a common pathophysiological basis, namely coronary atherosclerosis-based atheromatous plaque loosening, cracks, or rupture, so the intra-plaque substances that might result in thrombosis would be exposed in the blood, causing adhesion, activation, and aggregation of platelets on the damaged surface, followed by thrombosis as well as complete or incomplete occlusion of the diseased vessels. Cystatin C (CysC) and glomerular filtration rate (GFR) are indicators that could reflect renal dysfunction; the former is one of the sensitive indexes used to evaluate GFR [1] and could detect early renal damage. The important function of CysC is to regulate the hydrolysis of cysteine proteases; by inhibiting the activity of endogenous cysteine proteases, cell damage can be avoided. Recent studies showed that CysC participates in inflammatory reactions, production of vascular wall matrix, and homeostasis of degradation as well as the pathological processes of vascular injuries; it might also participate in regulating the formation, stabilization, and regression of atherosclerotic plaques [2]. Therefore, it would have predictive value regarding the prognosis of coronary artery disease (CAD) [3–5]. Meanwhile, CysC also exhibited predictive value regarding the morbidity and mortality of cardiovascular disease in people with normal renal function [6–8]. GFR equations based on CysC or a combination of CysC with creatinine may be superior to GFR equations based on creatinine alone in patients with CAD [9]. However, because the correlations of these two factors with ACS are rare, further study is still needed to determine whether CysC could be used to predict the severity of vascular disease. A number of studies about the correlations of CysC and CHD reported inconsistent conclusions. Therefore, this study aimed to further clarify the correlations of these two factors with vascular lesions and their severity in ACS.

## Methods

### Clinical data

Inclusion criteria: A total of 195 patients with ACS diagnosed and hospitalized in our hospital from July 2012 to June 2013 were enrolled. All the patients met the diagnostic criteria of ACS defined by the ACC/AHA in 2014 [10] and were confirmed by coronary angiography (CAG) to have stenosis ≥50% in at least one coronary artery. Another 39 patients with negative CAG results were selected during the same period for a total enrollment of 234 patients. This study was conducted in accordance with the declaration of Helsinki. This study was conducted with approval from the Ethics Committee of the North Huashan Hospital. Written informed consent was obtained from all participants.

Exclusion criteria: Patients were excluded if they had severe liver dysfunction (combined with jaundice, portal hypertension, ascites, and hepatic encephalopathy), severe renal dysfunction (estimated GFR [eGFR] < 30 mL/min/1.73 m^2^), acute cerebrovascular accident, acute infection, advanced cancer, a history of old myocardial infarction or structural heart diseases including cardiomyopathy or valvular disease, or a history of coronary stenting or coronary bypass surgery.

### Grouping

According to the results of CAG, the patients were divided into a single-vascular-lesion group (group S, 91 cases), a dual-vascular-lesion group (group D, 67 cases), and a multiple-vascular-lesion group (group M, 37 cases).

Count of CAS lesions: Stenosis ≥50% in any one of the three main coronary arteries (LAD, LCX, or RCA) was considered a significant lesion. Stenosis ≥50% of the left main stem was considered a dual-vascular lesion. Stenosis ≥50% of the intermediate branch was considered a single vascular lesion. Stenosis of a branch was considered a main vascular lesion. The sum of the diseased vessels with meaningful stenosis was then calculated.

### Baseline information

In addition to the baseline data, all the patients underwent electrocardiography (ECG) to calculate the left ventricular ejection fraction (LVEF) before and after admission (outpatient). The patients with STEMI and NSTEMI in each ACS subgroup were then categorized according to the Killip classification.

### ECG evaluation

A total of 187 patients completed ECG, with 152 completed within 48 h of admission using the Philips IE 33 ultrasound system. Thirty-five patients underwent ECG within 1 month of admission using the Philips HD 11 ultrasound system. LVEF was checked using M-mode ultrasonography and obtained using Simpson’s principle.

### Assessment of CAD severity

Assessment of CAD was performed according to the modified Gensini score [11].

### Killip classification

The Killip classification is a clinical classification of acute myocardial infarction-induced heart failure, and its classification standards were in accordance with the diagnostic criteria of ACS defined by the ACC/AHA in 2014 [10].

### Detection and grouping of serum CysC levels

A 4-mL sample of serum from each patient was subjected to a turbidimetric immunoassay to measure the concentration of serum CysC using was the Hitachi 7600-120 automatic biochemical analyzer; the kit was provided by Kyokuto (Tokyo, Japan). The normal reference value of CysC is ≤1.0 mg/L. The serum CysC concentration for all patients was measured within 24 h after admission.

CysC grouping: The CysC levels of all the hospitalized patients (234 cases) were divided into four subgroups according to the interquartile method: subgroup Q1, CysC < 1.1 mg/L (45 cases); subgroup Q2, 1.1 mg/L ≤ CysC < 1.2 mg/L (35 cases); subgroup Q3, 1.2 mg/L ≤ CysC < 1.5 mg/L (88 cases); and subgroup Q4, CysC ≥ 1.5 mg/L (66 cases).

### eGFR and grouping

The level of serum creatinine (Scr) was determined using a Hitachi automatic biochemical analyzer, and eGFR was calculated using the Cockcroft–Gault formula: Ccr (mL/min) = [(140 - age) × body weight (kg) × (0.85 (female)]/(72 × Scr [mg/dL]).

The eGFR levels of all the hospitalized patients (234 cases) were divided into three subgroups according to the degree of renal dysfunction: subgroup q1, moderate to severe renal dysfunction (30 mL/min ≤ eGFR < 60 mL/min) (44 cases); subgroup q2, mild to moderate renal dysfunction (60 mL/min ≤ eGFR < 90 mL/min) (94 cases); and subgroup q3, normal renal function (96 cases).

### Statistical analysis

The quantitative data are expressed as mean ± standard deviation. When homogeneity of variance existed among the groups, a t test was used to compare count data; otherwise, the t’ test was used. The CysC classification data are described as median (interquartile range), and the intergroup comparisons were made using the Wilcoxon rank sum test. Qualitative data are described as frequency (percentage), and the differences in CysC and eGFR in different vascular lesion subgroups were analyzed used the χ^2^ test. Correlations of CysC and eGFR with the Gensini score were analyzed using Pearson’s correlation coefficient. The relationship of each individual variable with ACS was then observed, and a single logistic regression model was established. To eliminate interference of confounding factors, a multivariate logistic regression model containing the main effects was established, and the predictive values of the variables with respect to the results were then analyzed. A multivariate logistic prediction model was established, with the inclusion criterion of *P* ≤ 0.05 and the exclusion criterion of *P* > 0.05. When the outcome variables were dichotomous, a nonconditional logistic regression model was established; when the outcome variables were ordered and categorical, an ordinal logistic regression model was established. Bilateral levels were set for the *P* values of all hypothetical tests, with the significance level set at 5% and the homogeneity of variance level was set at 10%. Statistical analysis was performed using STATA version 12.0 software.

## Results

### Index comparison

Compared with the control group, the proportion of male patients was significantly higher (*P* < 0.01), age was lower (*P* < 0.05), and the low-density lipoprotein cholesterol (LDL-C) level was higher (*P* < 0.01) in the STEMI group. Compared with the control group, the proportion of male patients was significantly higher (*P* < 0.01), the percentage of patients with diabetes was higher (*P* < 0.05), and the LDL-C level was higher (*P* < 0.05) in the NSTEMI group (Table 1).

**Table 1:** Baseline clinical baseline characteristic of all patients

		STEMI group (*n* = 77)	NSTEMI group (*n* = 35)	UAP group (*n* = 83)	Control group (*n* = 39)
Sex	Male	165 (84.42%)**	29 (82.86%)**	57 (68.67%)	20 (51.28%)
	Female	412 (15.58%)	6 (17.14%)	26 (31.33%)	19 (48.72%)
Age		59.19 ± 11.76*	67.14 ± 10.74	64.12 ± 10.36	64.59 ± 8.14
Hypertension		42 (54.55%)	25 (71.43%)	56 (67.47%)	23 (58.97%)
Diabetes		20 (25.97%)	14 (40.00%)*	25 (30.12%)	6 (15.38%)
LDL-C		3.11 ± 1.02**	3.07 ± 0.91*	2.65 ± 0.98	2.60 ± 0.77
Serum creatinine		74.78 ± 19.63	82.05 ± 24.91	75.55 ± 18.82	75.87 ± 19.73
Cys C		1.34 ± 0.48	1.46 ± 0.58	1.26 ± 0.27	1.30 ± 0.35
eGFR		99.47 ± 35.44	84.72 ± 35.42	83.03 ± 25.02	81.44 ± 23.95

Note: compared with control group

*P < 0.05

**P < 0.01

The comparison of different variables among different ACS subgroups (group S, D, and M) and the control group showed that the proportion of male patients in each ACS subgroup was higher than that of the control group, and the comparison among different ACS subgroups showed the results as group M > group S > group D (*P* < 0.05); the LDL-C level in each ACS subgroup was higher than that of the control group, and the comparison among different ACS subgroups showed the results as group D > group M > group S (*P* < 0.05); the CysC levels in groups D and M were higher than that of the control group, and the comparison among different ACS subgroups showed the results as group M > group D > group S (*P* < 0.05). Differences in age, proportion of patients with hypertension, proportion of patients with diabetes, LVEF, Killip grades, Scr level, and eGFR among the groups were not statistically significant (*P* > 0.05, Table 2).

**Table 2:** Variable comparison among different lesion groups

Index	Control (*n* = 39)	S (*n* = 91)	D (*n* = 67)	M ( *n* = 37)	*P*
Gender					0.0153
F	19 (48.72%)	20 (21.98%)	16 (23.88%)	8 (21.62%)	
M	20 (51.28%)	71 (78.02%)	51 (76.12%)	29 (78.38%)	
Age	63.59 ± 9.14	62.40 ± 10.56	61.67 ± 12.12	66.49 ± 10.70	0.1591
Hypertension	23 (58.97%)	53 (58.24%)	40 (59.70%)	30 (81.08%)	0.0670
LDL-C	2.60 ± 0.77	2.74 ± 1.07	3.10 ± 0.91	2.97 ± 1.03	0.0449
Diabetics	6 (15.38%)	27 (29.67%)	21 (31.34%)	11 (29.73%)	0.2608
LVEF	-	59.16 ± 5.17^a^	60.60 ± 5.67^b^	58.05 ± 8.00	0.0644
Killip classification					0.1090
Grade I	-	31 (77.50%)^c^	32 (86.49%)^d^	15 (68.18%)^e^	
Grade II	-	8 (20.00%)^c^	3 (8.11%)^d^	2 (9.09%)^e^	
Grade III	-	1 (2.50%)^c^	2 (5.41%)^d^	4 (18.18%)^e^	
Grade IV	-	0 (0.00%)^c^	0 (0.00%)^d^	1 (4.55%)^e^	
Cys C	1.30 ± 0.35	1.27 ± 0.30	1.34 ± 0.44	1.51 ± 0.59	0.0296
Scr	75.87 ± 19.73	73.29 ± 14.82	77.87 ± 21.31	80.84 ± 29.00	0.2370
eGFR	81.44 ± 23.95	92.31 ± 31.02	90.05 ± 32.50	83.56 ± 34.39	0.2146

Note:

^a^the cases with valid LVEF data in group S were 88 cases

^b^the cases with valid LVEF data in group D were 62 cases

^c^the cases with valid Killip classification data in group S were 40 cases

^d^the cases with valid Killip classification data in group D were 37 cases

^e^the cases with valid Killip classification data in group M were 22 cases

### Relationships between different variables and vascular lesions

Using a univariate logistic regression model while not controlling other factors, each variable was evaluated regarding correlation with vascular lesions. The results showed that the gender composition (male) and LDL-C and CysC levels were positively correlated with vascular lesions (*P* < 0.05), but the correlations of eGFR and other variables did not reach statistical significance (*P* > 0.05) (Table 3).

**Table 3:** Univariate logistic regression analysis of different variables with vascular lesions

Factor	Coefficient	SD	Statistic	*P*	OR (95%CI)
M	0.6362047	0.2794	2.28	0.0230	1.8893 (1.0927–3.2667)
Age	0.0080341	0.0108	0.75	0.4560	1.0081 (0.9870–1.0296)
Hypertension	0.4309775	0.2446	1.76	0.0780	1.5388 (0.9528–2.4851)
LDL-C	0.3062137	0.1254	2.44	0.0150	1.3583 (1.0623–1.7367)
Diabetics	0.3487115	0.2616	1.33	0.1830	1.4172 (0.8487–2.3666)
Cys C	0.7297879	0.3015	2.42	0.0150	2.0746 (1.1490–3.7459)
Scr	0.0103784	0.0061	1.70	0.0890	1.0104 (0.9984–1.0226)
eGFR	0.0002985	0.0038	0.08	0.9370	1.0003 (0.9929–1.0078)

Using a multivariate logistic regression model while controlling other factors, each variable was analyzed regarding correlation with vascular lesions. The results showed that CysC level, gender composition (male), LDL-C level, and hypertension were positively correlated with vascular lesions (*P* < 0.05). For the CysC level, the odds ratio was 2.09, indicating that when CysC was increased by 1 mg/L, the risk of an additional vascular lesion increased by 2.09 times. Because the *P* value of eGFR in the univariate regression analysis was too high (0.937), it was not included in the multivariate statistical regression model (Table 4).

**Table 4:** Multivariate logistic regression analysis of different variables with vascular lesions

Factor	Coefficient	SD	Statistic	*P*	OR (95%CI)
Cys C	0.736095	0.3095	2.38	0.0170	2.0878 (1.1382–3.8295)
Male	0.776684	0.2928	2.65	0.0080	2.1743 (1.2250–3.85920)
LDL-C	0.329243	0.1270	2.59	0.0100	1.3899 (1.08360–1.7828)
Hypertension	0.514262	0.2565	2.00	0.0450	1.6724 (1.0115–2.7650)

### Linear relation of CysC and eGFR with Scr values

**Fig. 1:**
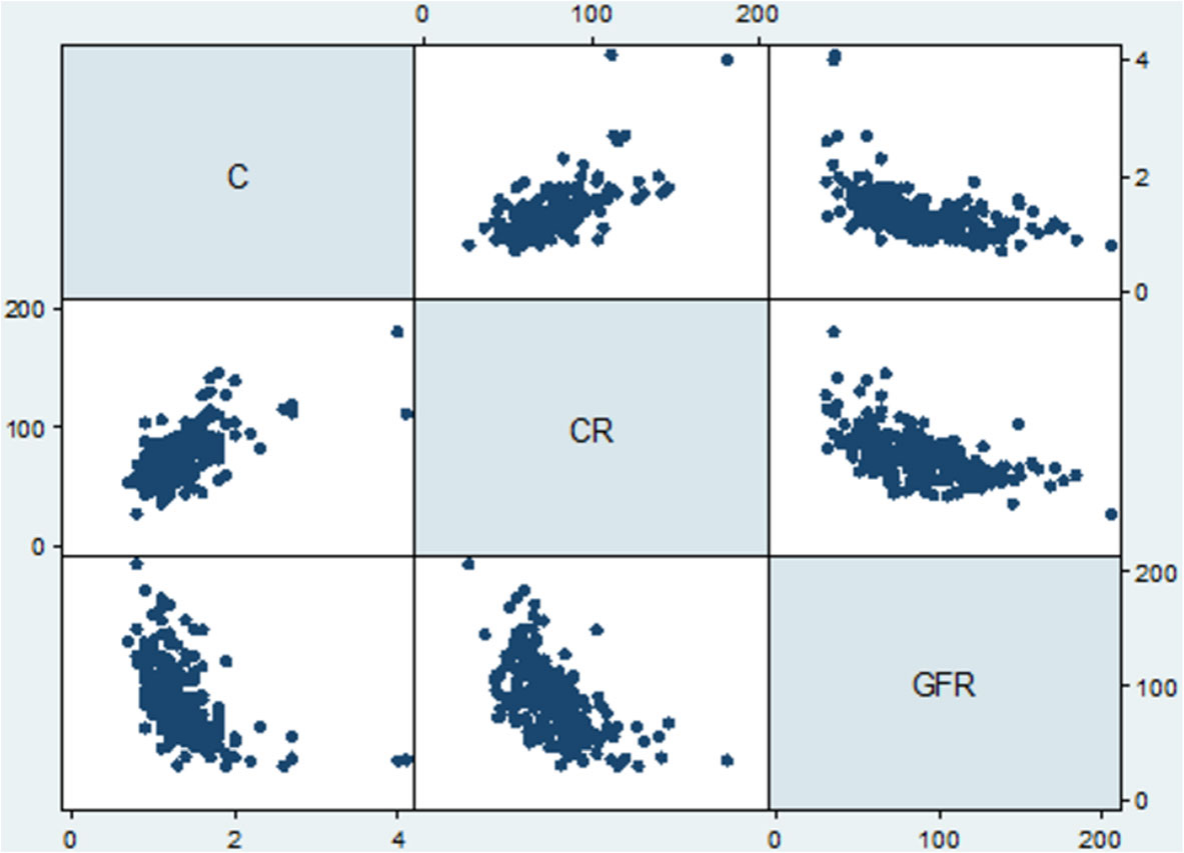
Scatterplots showed linear relationships between serum cystatin C, eGFR and Scr

CysC and Scr levels were measured in 234 patients after they were hospitalized, and eGFR values were calculated. A positive correlation was detected between CysC and Scr values based on Pearson’s correlation coefficient (*r* = 0.6158, *P* < 0.0001), and a negative correlation was detected between eGFR and CysC level (*r* = -0.5115, *P* < 0.0001) and between eGFR and Scr level (*r* = -0.5545, *P* < 0.0001) (Fig. 1).

### Relationships of CysC level and kidney function with vascular lesions

By comparing the constitute ratio differences of different CysC levels (four subgroups) and different eGFR levels (three subgroups) in different vascular lesion groups, the correlations of different CysC levels and kidney dysfunction with vascular lesions were determined. Among the CysC subgroups, the constitute ratios in subgroup Q1 (low CysC concentration) were group S > group D > group M and those in subgroup Q4 (high CysC concentration) were group M > group D > group S; these differences were statistically significant (*P* < 0.05). Among the different eGFR subgroups, the constitute ratios in subgroup q1 (moderate to severe renal dysfunction) were group M > group D > group S, those in subgroup q2 (mild to moderate renal dysfunction), and those in subgroup q3 (normal renal function) were group S > group D > group M, but the differences were not statistically significant (*P* > 0.05) (Table 5).

**Table 5:** Comparison of constitute ratio differences of different Cys C subgroups and different eGFR subgroups in different vascular lesion groups

Index		S	D	M	Statistic	*P*
Cys C	Q1	23 (25.27%)	10 (14.93%)	4 (10.81%)	Z = 7.25	0.0267
	Q2	12 (13.19%)	13 (19.40%)	5 (13.51%)		
	Q3	35 (38.46%)	26 (38.81%)	10 (27.03%)		
	Q4	21 (23.08%)	18 (26.87%)	18 (48.65%)		
eGFR	q1	13 (14.29%)	12 (17.91%)	10 (29.73%)	Z = 2.90	0.2342
	q2	36 (39.56%)	26 (38.81%)	13 (35.14%)		
	q3	42 (46.15%)	29 (43.28%)	13 (35.14%)		

### Comparison of CysC medians

**Fig. 2:**
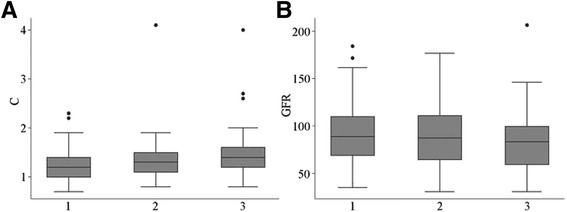
**a** Correlation of Cys C median with different vascular disease variables by box-plot; Note: Abscissa 1 represented group S, 2 represented group D, 3 represented group M, the ordinate represented the Cys C value, and the transverse lines in the blocks represented the medians. **b** Correlation of eGFR median with different vascular disease variables by box-plot. Note: Abscissa 1 represented group S, 2 represented group D, 3 represented group M, the ordinate represented the eGFR value, and the transverse lines in the blocks represented the medians

According to the CysC ranges and medians in different vascular lesion subgroups, a box plot could be drawn (Fig. 2a). As seen from the box plot, with an increase in vascular lesions, the median CysC level showed an increasing trend. The Spearman rank correlation coefficient between these two was 0.1576, which reached statistical significance (*P* = 0.0278). Quantile regression analysis showed that the median CysC level was increased on average by 0.1 mg/L for each additional vascular lesion (*P* = 0.513) (Fig. 2a).

### Comparison of eGFR medians

According to the eGFR ranges and medians in different vascular lesion subgroups, a box plot could be drawn (Fig. 2b). As seen from the box plot, with an increase in vascular lesions, the median eGFR showed a decreasing trend. The Spearman rank correlation coefficient between these two was -0.0993, which did not reach statistical significance (*P* = 0.1671). Quantile regression analysis showed that the median eGFR was decreased on average by 2.0 for each additional vascular lesion (*P* = 0.086) (Fig. 2b).

### Correlation comparison of different vascular lesions and severity

By comparing the differences in Gensini scores among the different vascular lesion subgroups, the correlations of vascular lesions with Gensini scores were determined. Gensini scores showed differences among different subgroups, with group M > group D > group S, and the differences were statistically significant (67.32 ± 40.04 vs. 43.78 ± 25.50 vs. 30.20 ± 22.92, *P* < 0.0001).

### Correlations of different CysC levels with vascular lesion severity

By comparing the differences of Gensini scores among different CysC subgroups, the correlations of CysC levels with Gensini scores were then determined. The results showed that the Gensini scores showed differences among different CysC subgroups, subgroup Q4 > subgroup Q 3 > subgroup Q2 > subgroup Q1, and the differences were statistically significant (50.00 ± 37.37 vs. 42.67 ± 30.40 vs. 39.13 ± 22.58 vs. 30.24 ± 22.25 mg/L, *P* < 0.05).

### Correlations of different renal functions with vascular lesion severity

By comparing the differences of Gensini scores among different eGFR subgroups, the correlations of different renal functions with Gensini scores were then determined. The results showed that the Gensini scores showed differences among different eGFR subgroups, subgroup q3 > subgroup q1 > subgroup q2, but the differences were not statistically significant (43.2 ± 29.84 vs. 41.54 ± 35.65 vs. 40.91 ± 30.97 mL/min/1.73 m^2^, *P* > 0.05).

### Linear relationships of serum CysC level, eGFR, and Gensini score

**Fig. 3:**
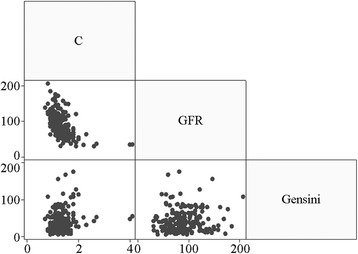
Scatterplot matrix among Cys C, eGFR, and Gensini score in patients with ACS. The pair Pearson correlation coefficients among the above three indexes

Pearson’s correlation coefficient was used to calculate the paired linear relationships among CysC level, eGFR, and Gensini score. CysC was negatively correlated with eGFR (*r* = -0.5073, *P* < 0.0001) but positively correlated with Gensini score (*r* = 0.1811, *P* < 0.05); eGFR and Gensini score were positively correlated (*r* = 0.0738, *P* > 0.05) (Fig. 3).

### Correlations of different Killip grades with vascular lesion severity

By comparing the differences in Gensini scores among the different Killip classification subgroups, the correlations of Killip grades with Gensini scores were then determined. Gensini scores showed differences among different Killip classification subgroups, with Killip grade IV (one case) > Killip grade II > Killip grade III > Killip grade I, but the differences were not statistically significant (168.00 ± 0.00 vs. 67.15 ± 39.32 vs. 51.57 ± 16.78 vs. 46.36 ± 26.53, *P* > 0.05).

## Discussion

The purpose of this study was to investigate the correlations of serum CysC level and eGFR with vascular lesions and their severity in patients with ACS. In recent years, some scholars suggested that inflammation and immune responses were the intermediary and key factors of cardiovascular remodeling and onset [12]. Cardiovascular events such as acute myocardial infarction are related to the inflammatory activities of lesions and matrix remodeling induced by plaque rupture. Optical coherence tomography is a feasible and safe imaging modality for patients with CAD and allows identification of the various microstructures of the atherosclerotic plaque such as plaque rupture, thin-cap fibroatheroma, lipid core, and intracoronary thrombus [13]. Many studies observed that the weakening process of fibrous caps mainly resulted from extracellular matrix degradation, which was caused by activated proteases such as matrix metalloproteinases and serine proteases released by the activated macrophages and smooth muscle cells in the plaques [14, 15]. Recently, two cysteine proteases that could promote the dissolving of elastic tissues, cathepsin S and cathepsin K, were found to be overexpressed in atherosclerotic lesions [16]. The main functions of serum CysC are to inhibit the activity of endogenous cysteine proteases, to regulate the intracellular metabolism of proteins [17], and to play the role of inflammatory mediators through activating neutrophils [18]; its mechanisms are involved in inhibiting inflammatory factors, confronting plasminogens, regulating the activity of pre-hormones, and regulating proteinases inside and outside cells [19–21]. Studies have shown that the serum CysC level was related to the severity of ACS [22]. Tebaldi [23] found that a reduced CrCl value (45 mL/min) was independently associated with a significantly lower incidence of positive FFR (0.80 or less). Regarding correlations between coronary vascular lesions and the severity of coronary vascular diseases, Corpus [24] found that the incidence of major adverse cardiac events such as death, repeated myocardial infarction, or repeated revascularization in patients with acute myocardial infarction plus multiple vascular lesions during the 1-year follow-up period was significantly higher than in patients with a single vascular lesion. Their results suggested that the ACS patients with multiple vascular lesions had more severe disease and a worse prognosis.

The analysis of correlations between CysC level and vascular lesions in the ACS patients showed the CysC levels in group D and M were higher than those in the control group, and the comparison among different ACS subgroups showed group M > group D > group S (group M 1.51 ± 0.59 mg/L vs. group D 1.34 ± 0.44 mg/L vs. group S 1.27 ± 0.30 mg/L vs. control group 1.30 ± 0.35 mg/L, *P* = 0.0296). The single-factor regression analysis of the vascular lesions in these ACS patients suggested that the CysC level was positively correlated with the vascular lesions (*P* = 0.0150), and the multifactor regression analysis showed the same result (*P* = 0.017); among different CysC subgroups, the constitute ratios in the lower-quartile groups were group S > group D > group M, and those in the high-quartile groups were group M > group D > group S, and the differences were statistically significant (*P* < 0.0267). It could be seen from the box plot (Fig. 2a) that with increasing vascular lesions, the median CysC level also showed an increasing trend. The Spearman rank correlation coefficient of these two was 0.1576 (*P* = 0.0278).

The analysis of correlations between CysC level and vascular lesion severity in the ACS patients showed that the Gensini scores exhibited differences among different CysC subgroups, with subgroup Q4 (high-quartile group, 50.00 ± 37.37 mg/L) > subgroup Q3 (42.67 ± 30.40 mg/L) > subgroup Q2 (39.13 ± 22.58 mg/L) > subgroup Q (lower-quartile group) 30.24 ± 22.25 mg/L (*P* = 0.0286). Using the scatterplot matrix of CysC, eGFR, and Gensini scores, Pearson’s correlation coefficient calculation showed that CysC was positively correlated with the Gensini score (*r* = 0.1811, *P* < 0.05).

The results of this study suggested that CysC level was positively correlated with the vascular lesions and their severity in these ACS patients, and the differences were statistically significant, consistent with the results of Jernberg [22]. Meanwhile, the comparison between the vascular lesions and Gensini scores in the ACS patients showed that group M (67.32 ± 40.04 mg/L) > group D (43.78 ± 25.50 mg/L) > group S (30.20 ± 22.92 mg/L) (*P* < 0.0001), fully consistent with the results of Corpus [24].

The inconsistent results regarding CysC mainly derives from the fact that CysC is just one member of the cysteine protease inhibitors, and CysC and its fragment can regulate inflammatory processes and participate in the pathological physiological process of atherosclerotic plaque formation. CysC may inhibit the activity of cathepsin S and K, reduce degradation of blood vessels and vascular remodeling, and postpone the occurrence and development of atherosclerosis. Patients with increased CysC levels may have higher cardiac mortality, and the mortality of patients with higher CysC levels was 3.87-fold higher than that in patients with lower CysC levels. Sai E et al. [25] found that increased CysC levels can be a predictive factor of recurrence of cardiovascular events, which may be derived from the fact that CysC contributes to the pathological physiological process of atherosclerosis and inflammation. However, there are still multiple contradictions with respect to the relationship between CysC and CHD. For these contradictions, Noto et al. [26] believed that the difference is mainly caused by a negative acute-phase reaction during acute myocardial infarction. The case of a patient with acute myocardial infarction who had normal expression levels of CysC after 7 days in the hospital also supports this hypothesis. However, another hypothesis indicated that the expression of CysC is reduced before acute cardiovascular events, which further activates inflammatory factors and cell factors and induces a lower level of CysC. One study showed that transforming growth factor β1 (TGF-β1) is an important factor inducing CysC [27]; serum TGF-β1 levels are significantly reduced in atherosclerosis, and increased TGF-β1 can inhibit the process of lesion formation. CysC levels are near normal in the recovery phase, and may even be increased, which is also caused by regulation of inflammatory and cell factors, body compensatory mechanisms, and the gradual repair and conserved result of inflammatory patches. Luc et al. [28] found that ischemic events may be caused by a relative shortage of proteinase inhibitors (such as CysC). Although the inflammatory process increased the expression of CysC, CysC is still not enough than increased protease in inflammatory atheromatous plaques.

Collectively, many clinical studies have shown that CysC is related to CHD. Patients with ACS with vascular lesions should be further studied owing to the limited number of samples in this study. Simultaneously, determining whether CysC can be used as a potential biomarker for prognostic evaluation and prediction of major adverse cardiac events and has potential guiding significance and value for revascularization will be the next research direction.

## Conclusions

Serum CysC levels might reflect the severity of vascular lesions in patients with ACS, and high CysC levels had a predictive value regarding the severity of vascular lesions in ACS.

## Abbreviations

ACS: Acute coronary syndrome
CAD: Coronary artery disease
CAG: Coronary angiography
CHD: Coronary heart disease
CysC: Cystatin C
GFR: Glomerular filtration rate
LVEF: Left ventricular ejection fraction
NSTEMI: Non-ST segment-elevated myocardial infarction
STEMI: ST segment-elevated myocardial infarction
